# *Capparis* L. (*Capparaceae*): A Scoping Review of Phytochemistry, Ethnopharmacology and Pharmacological Activities

**DOI:** 10.3390/molecules30183705

**Published:** 2025-09-11

**Authors:** Bashaer Alsharif, Fabio Boylan

**Affiliations:** 1 Department of Pharmaceutical Sciences, Faculty of Pharmacy, Umm Al-Qura University, Makkah 21955, Saudi Arabia; alsharib@tcd.ie; 2 School of Pharmacy and Pharmaceutical Sciences, Trinity Biomedical Sciences Institute and NatPro Centre, Trinity College Dublin, D02 PN40 Dublin, Ireland

**Keywords:** *Capparis*, ethnopharmacology, phytochemistry, pharmacological activity

## Abstract

Capers (*Capparis* L.), a genus of shrub-like plants within the family Capparaceae, exhibit remarkable ecological adaptability and have long been used in traditional medicine, food, and agroforestry. Phytochemical investigations have identified a wide array of bioactive compounds—including flavonoids, alkaloids, glucosinolates, spermidine derivatives, and other unique secondary metabolites—particularly in species such as *C. spinosa* and *C. decidua*. Pharmacological studies have reported diverse biological activities, including antimicrobial, anti-inflammatory, immunomodulatory, antidiabetic, and antioxidant effects. This review provides a comprehensive overview of the genus, with particular attention to its botanical characteristics, ethnomedicinal relevance, phytochemical composition, and pharmacological potential.

## 1. Introduction

The genus *Capparis* L. (Capparaceae) comprises between 250 and 400 species of flowering plants, distributed primarily across tropical and subtropical regions of Africa, Asia, the Mediterranean, and the Americas, with some species extending into temperate areas such as Southern Europe and Southwest Asia [1,2]. It belongs to the family Capparaceae, which consists of approximately 45 genera and over 700 species, primarily found in arid and semi-arid environments [3].

*Capparis* species are highly adaptable, thriving in diverse habitats such as rocky cliffs, deserts, and tropical forests. Many species are deeply integrated into traditional cultural practices, where they serve as sources of food, herbal remedies, and materials in agroforestry. Particularly, the flower buds of *Capparis spinosa* (capers) are consumed as pickled condiments [4]. In addition to their ethnobotanical importance, *Capparis* plants also contribute to ecosystem functions such as erosion control and pollinator support—though these roles remain relatively underexplored [5].

With the increasing amount of research on *Capparis* species, there is now an opportunity to create a comprehensive overview that brings together both earlier foundational work and recent findings. Previous studies remain important for understanding the genus’s taxonomy, chemistry, and traditional use [5,6,7,8,9], while newer research using advanced analytical methods and biological models has added important details about their chemical diversity and biological activities. This review integrates existing knowledge and recent findings to provide a broad and updated overview of the genus *Capparis* and its potential.

## 2. Botanical Classification, Morphology, and Geographical Distribution

The family is traditionally divided into two subfamilies: Capparidoideae, primarily shrubs or trees with fleshy, indehiscent fruits (including *Capparis*), and Cleomoideae, mainly herbaceous plants with unilocular capsules (siliques) as fruits [10].

Typical *Capparis* species are shrubs or small bushes, reaching 30–100 cm in height with deep root systems. Leaves are alternate, simple, and oval to elliptic, sometimes leathery, often bearing spiny stipules. Flowers are hermaphroditic, 5–7 cm wide, with four purplish sepals and white petals, usually borne singly in leaf axils. Stamens are numerous and radiate beyond the petals, attached at the base of a gynophore. The fruit, known as a caperberry, is ellipsoid with thin skin that ruptures when ripe, revealing kidney-shaped seeds embedded in pale crimson flesh [8].

**Figure 1:**
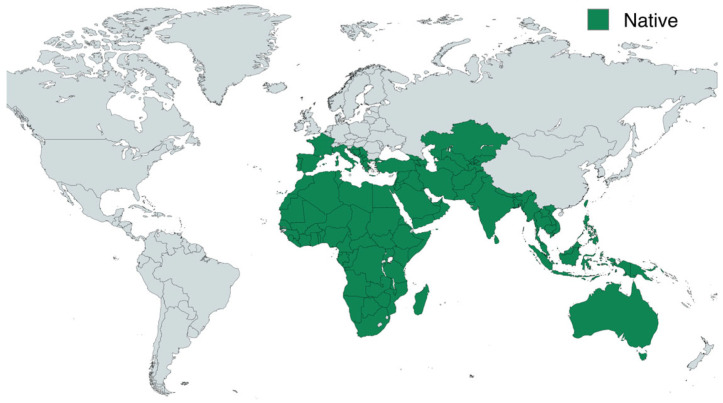
Natural distribution of caper based on data from Kew’s Plants of the World Online (POWO) [11].

The distribution of *Capparis* species shows broad geographic patterns across tropical, subtropical, and temperate regions. Most species occur in Africa, the Arabian Peninsula, South and Southeast Asia, and Australia, with some extending to the Mediterranean basin and parts of Central Asia. Widely known species such as *C. spinosa* and its subspecies dominate the Mediterranean and adjacent regions, while others, such as *C. decidua* and *C. sepiaria*, are more common in arid and semi-arid zones of Africa and Asia. The genus also extends to tropical areas, including Malesia and northern Australia, reflecting its adaptability (Figure 1) [11].

## 3. Ethnopharmacology

**Figure 2:**
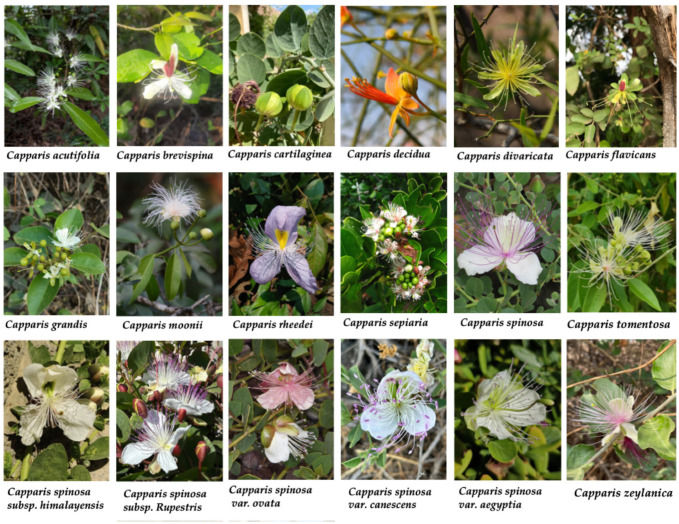
Representative species of the genus *Capparis* (Capparaceae). Images sourced from GBIF/iNaturalist; full attributions are provided in Supplementary Table S1.

The genus *Capparis* has long held significance in traditional medicine across Asia, Africa, and the Mediterranean. Various species have been employed to manage a wide range of ailments, including inflammatory conditions, digestive and respiratory disorders, metabolic diseases, and infections. These practices highlight the cultural and therapeutic importance of the genus, reflecting its diverse bioactive potential. Table 1 provides an overview of some of the most commonly used *Capparis* species (Figure 2), their geographic distribution, plant parts used and reported ethnomedicinal applications.

## 4. Phytochemistry

Several studies have investigated and quantified the chemical and bioactive constituents of different organs of *Capparis* species, including roots, seeds, leaves, buds, and fruits. The chemical composition is known to vary due to factors such as genotype, harvesting time, plant part size, environmental conditions, and preservation methods [5]. The major classes of phytochemicals reported from various *Capparis* species include alkaloids, flavonoids, fatty acids, glucosinolates, and sterols.

### 4.1. Alkaloids

**Figure 3:**
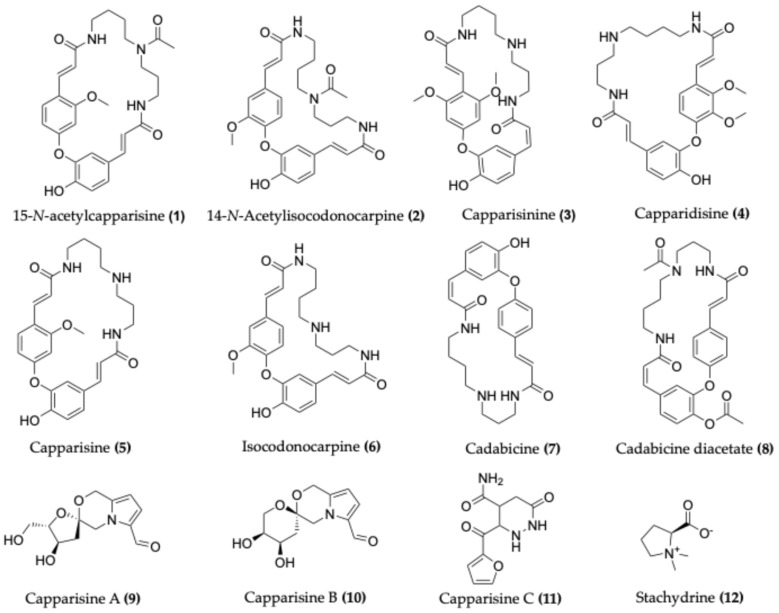
Chemical structures of selected alkaloids found in various *Capparis* species.

Alkaloids are a prominent class of nitrogenous compounds in *Capparis*, distributed throughout various plant parts but especially abundant in the roots. The following section summarises key alkaloids identified in different *Capparis* species, illustrating the chemical diversity (Figure 3) within the genus [5]. For example, the roots of *C. decidua* contain compounds such as 15-*N*-acetylcapparisine (**1**), 14-*N*-Acetylisocodonocarpine (**2**), capparisinine (**3**), capparidisine (**4**), capparisine (**5**), and isocodonocarpine (**6**) [43,44].

*C. spinosa* roots and fruits have yielded cadabicine (**7**), cadabicine diacetate (**8**), capparispine, capparispine 26-O-β-D-glucoside, and cadabicine 26-O-β-D-glucoside hydrochloride [45,46]. Additional pyrrole alkaloids isolated from the fruits of *C. spinosa* include capparisine A (**9**), capparisine B (**10**), capparisine C (**11**), 2-(5-hydroxymethyl-2-formylpyrrol-1-yl) propionic acid lactone, and *N*-(3′-maleimidyl)-5-hydroxymethyl-2-pyrrole formaldehyde [47].

The roots of *Capparis aphylla* (*syn. C. decidua*) have yielded alkaloids like cappariline, capparine, and capparinine [48,49,50]. From the whole plant of *C. himalayensis*, three further alkaloids, capparin A, B, and C, have been reported [34]. Stachydrine (**12**), a proline-derived alkaloid, has also been isolated from the genus and is characterised by its quaternary ammonium structure, which underlies its diverse bioactivities [51].

Recent studies have extended the known alkaloid spectrum of the genus. For instance, two novel spermidine alkaloids, acutifoline A and B, were isolated and structurally characterised from *C. acutifolia* roots [12,13]. Additionally, new alkaloids from *C. spinosa* fruits include 6-hydroxy-2′-(methylthio)-4′H-spiro[indoline-3,5′-thiazol]-2-one, 2-(hydroxythio)-6-methoxy-1-methyl-1H-indole-3-carbaldehyde [52] and more recently, (2*S*,3*S*)-4-(5,6-dimethylpyrazin-2-yl) butane-1,2,3-triol [53].

### 4.2. Flavonoids

**Figure 4:**
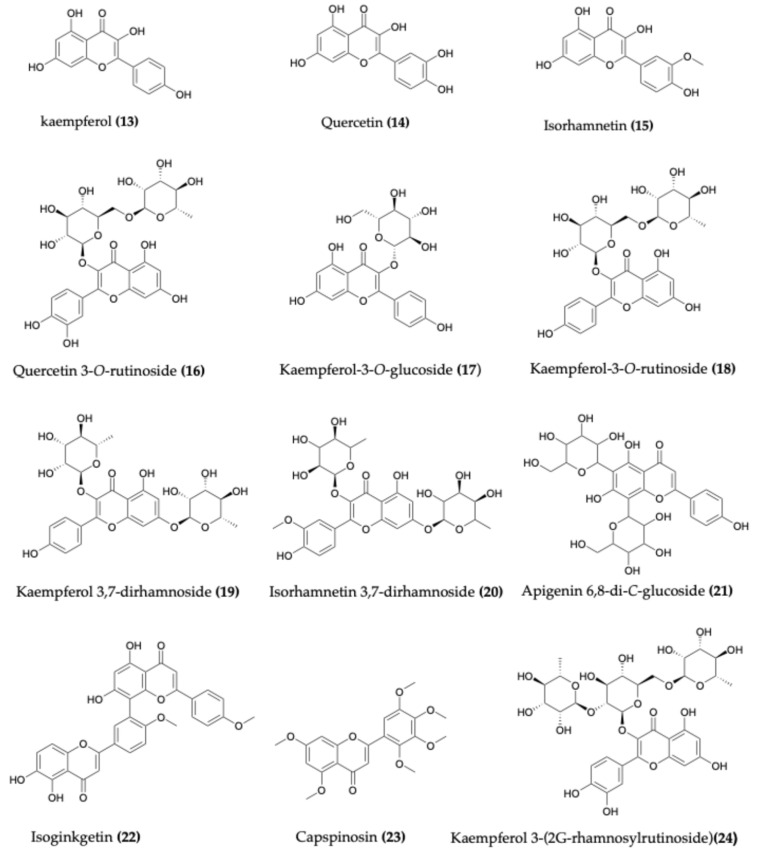
Chemical structures of selected flavonoids identified in *Capparis* species.

Flavonoids are a diverse class of plant secondary metabolites responsible for a wide range of pigmentation, from yellow to blue, in various plant organs. In *C. spinosa*, the flavonoids contribute to the characteristic yellow coloration of fruits and buds. These compounds occur both as aglycones and as glycosides (Figure 4), where sugar moieties influence their solubility and stability. Flavonoids include mono-flavonoids as well as biflavonoids, which contain two flavone units. This widespread distribution and chemical diversity highlight their importance in the genus *Capparis* [5].

Flavonoids are among the most extensively studied compounds in *C. spinosa*, especially in its leaves, flower buds, and fruits [5]. Identified compounds include kaempferol (**13**), quercetin (**14**), isorhamnetin (**15**), quercetin 3-*O*-rutinoside (**16**), rhamnocitrin, kaempferol-7-*O*-rhamnoside, kaempferol-3-*O*-rhamnorutinoside, kaempferol-3-*O*-glucoside (**17**), kaempferol-3-*O*-rutinoside (**18**), and kaempferol-3-*O*-rhamnosylrutinoside. Other flavonoids include kaempferol 3,7-dirhamnoside (**19**), quercetin-7-*O*-rutinoside, isoquercetin, quercetin-3-*O*-glucoside-7-*O*-rhamnoside, isorhamnetin 3-*O*-rutinoside, and isorhamnetin 3,7-dirhamnoside (**20**). Additionally, apigenin, apigenin 8-*C*-glucoside, apigenin 6,8-di-*C*-glucoside (**21**), thevetiaflavone, isoginkgetin (**22**), ginkgetin, sakuranetin, and capspinosin (**23**), a distinctive flavonoid specific to *C. spinosa*, have also been reported [54].

Other *Capparis* species such as *C. tweediana*, *C. atamisquea*, *C. cynophallophora*, *C. humilis*, *C. retusa*, and *C. speciosa* contain flavonoids kaempferol, quercetin, kaempferol-3-*O*-rutinoside and kaempferol-3-*O*-rhamnosylrutinoside in their leaves [55]. The aerial parts of *C. himalayensis* have yielded compounds flavonoids kaempferol, quercetin 3-*O*-rutinoside, and rhamnocitrin [34]. Similarly, *C. decidua* contains glucosides kaempferol, quercetin, and rhamnocitrin as well as flavonoids kaempferol 3,7-dirhamnoside, quercetin-7-*O*-rutinoside [56,57,58]. Additionally, the roots of *C. tenera* have been reported to contain acacetin-7-*O*-rutinoside [59]. In a recent study, several glycosylated flavonoids were isolated from the leaves of *C. cartilaginea*, including kaempferol 3-(2G-rhamnosylrutinoside) (**24**), quercetin 3-(2G-rhamnosylrutinoside), quercetin 3-neohesperidoside, kaempferol 3-neohesperidoside, quercetin 3-*O*-rutinoside, kaempferol-3-*O*-rutinoside and isorhamnetin 3-*O*-rutinoside [60].

### 4.3. Fatty Acids

**Figure 5:**
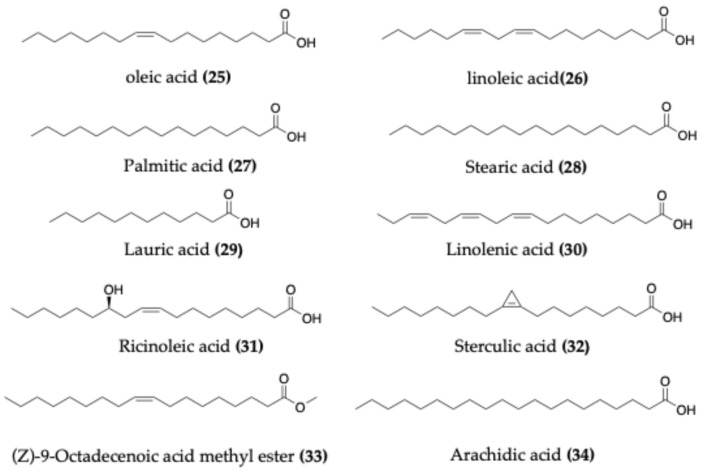
Chemical structures of selected fatty acids identified in *Capparis* species.

Species of *Capparis* are known for their lipid-rich seeds, which typically contain 10–15% fat in the form of triacylglycerols. Fatty acid profiling has identified both saturated and unsaturated fatty acids (Figure 5). Common constituents include oleic acid (**25**), linoleic acid (**26**), palmitic acid (**27**), stearic acid (**28**), lauric acid (**29**), linolenic acid (**30**), and myristic acid [5]. Remarkably, *C. zeylanica* seed oil contains up to 30% ricinoleic acid (**31**), a compound also known from castor oil and widely studied for its physicochemical properties [61]. In addition, unusual fatty acids such as sterculic acid (**32**) and malvalic acid have also been reported in the seeds of *Capparis* species [62].

Further profiling across species has confirmed these results. For example, seeds of *C. spinosa* and *C. decidua* contain oleic, linoleic, palmitic, and stearic acids, along with (Z)-9-octadecenoic acid methyl ester (**33**) [63,64,65]. *C. aphylla* seeds have yielded arachidic acid (**34**) [66], while *C. zeylanica* additionally contains sterculic and malvalic acids [62,67]. Palmitoleic acid was identified in the seeds of *C. divaricata* [68], and oleic acid has also been reported in the seeds of *C. ovata* [69].

### 4.4. Glucosinolates

Glucosinolates are sulphur- and nitrogen-containing secondary metabolites, comprising a glucose moiety bound to a sulphur-derived amino acid. They are predominantly found in species belonging to the order Brassicales (also referred to as Capparales) and are responsible for the well-known “mustard oil bomb” defence mechanism. Upon tissue disruption, glucosinolates are hydrolysed by myrosinase enzymes to release pungent isothiocyanates, which contribute to the distinctive aroma of capers and exhibit notable antioxidant and chemopreventive activities [5,70].

**Figure 6:**
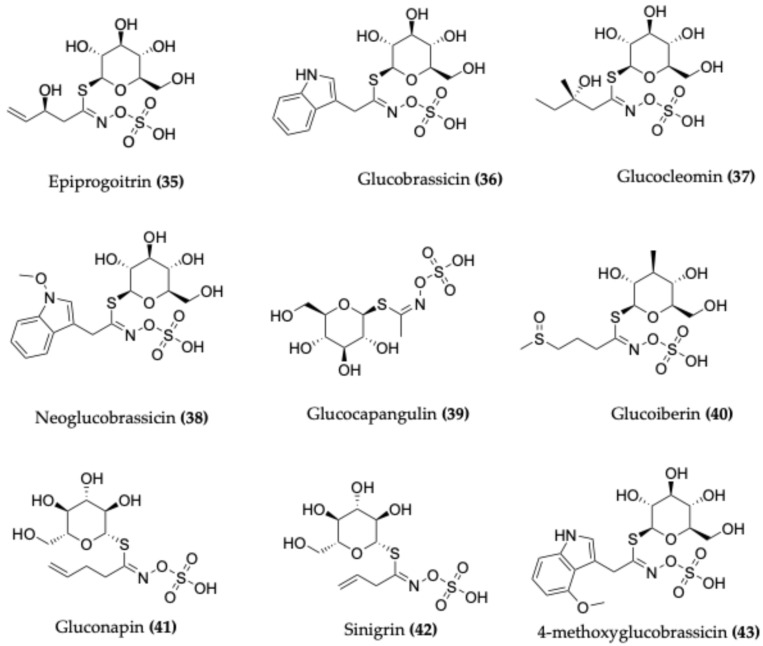
Chemical structures of selected glucosinolates identified in *Capparis* species.

Several *Capparis* species have been reported to contain a wide array of glucosinolates (Figure 6). In *C. ovata*, epiprogoitrin (**35**) and glucobrassicin (**36**) have been detected in the leaves, seeds, flowers, and young shoots [71]. The variety *C. ovata* var. *palaestina* contains glucocapangalin, glucocleomin (**37**), and neoglucobrassicin (**38**) in its leaves [72]. Investigations into Egyptian Capparis species have also identified glucoiberin, glucocapparin, sinigrin, glucocleomin, glucobrassicin, and glucocapangulin (**39**) [32].

In another study, *C. ovata* was reported to possess several additional glucosinolates, including glucoiberin (**40**), gluconapin (**41**), sinigrin (**42**), glucocapparin, progoitrin, and glucosinalbin in its leaves, seeds, flowers, and flower buds [71]. In *C. spinosa*, 4-methoxyglucobrassicin (**43**) has been identified in the leaves [73], and *C. linearis* Jacq. contains 3-methyl-3-butenylglucosinolate in the leaves [74]. In *C. cartilaginea*, the glucosinolates glucoputranjivin and butyl glucosinolate have been identified in the leaves [60]. A recent study showed that is the main glucosinolate in *C. spinosa*, *C. spinosa* subsp. *rupestris*, and *C. orientalis*, but was not detected in *C. richardii*. The study also reported three uncommon glucosinolates, glucocochlearin, glucohirsutin, and glucoarabin, in *Capparis* species for the first time [75].

### 4.5. Sterols

Sterols—oxygen-containing steroidal alcohols—are typically found in the unsaponifiable fraction of plant oils and constitute a minor yet functionally significant component. In *Capparis* species, seed oils can comprise up to approximately 15% of the dry weight, with sterols accounting for roughly 1–2% of total lipids [5].

**Figure 7:**
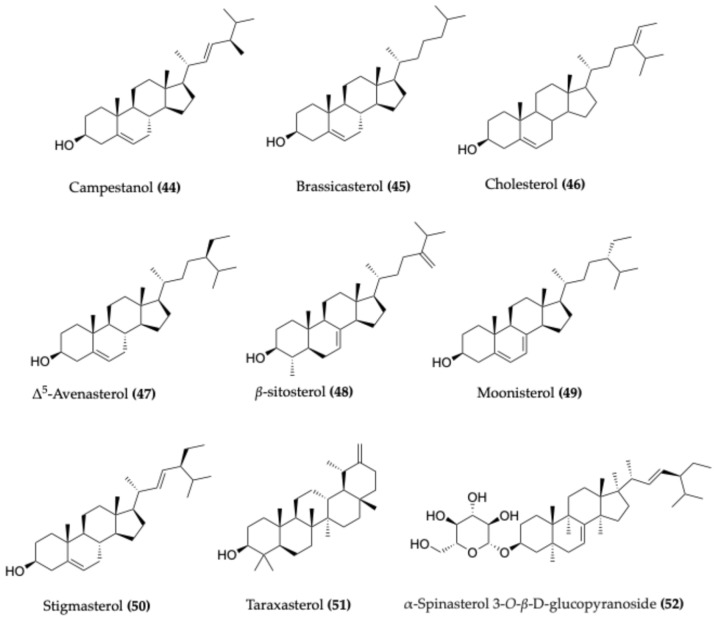
Representative sterols identified in *Capparis* species.

A diverse range of sterols has been identified across various *Capparis* species (Figure 7), particularly in seeds and aerial parts. Campestanol (**44**) and brassicasterol (**45**) were detected in *C. spinosa* seed oil, the latter at 3.39 mg/kg [76]. Cholesterol (**46**), a minor sterol in *C. spinosa* seed oil, was also isolated from the root bark of *C. corymbosa* [77]. Δ^5^-Avenasterol (**47**) accounted for 6% of sterols in *C. spinosa* seed oil, with concentrations ranging from 138.8 to 599.4 mg/kg in both *C. spinosa* and *C. ovata* [69,76].

Among the most abundant sterols, β-sitosterol (**48**) represented 57.53% of total sterols in *C. spinosa* seed oil (1390 mg/kg), and has been widely reported in other plant parts of *C. spinosa*, *C. decidua*, *C. aphylla*, *C. sepiaria*, *and C. moonii*, where it exhibited notable anti-inflammatory effects [76,78].

Additional sterols include 24-*β*-methylcholest-7-ene-22-one-3*β*-ol and 24-*β*-methylcholest-9(11)-ene-22-one-3α-ol, both identified in the alcoholic extract of *C. decidua* root bark [79], and moonisterol (**49**) from the fruit of *C. moonii* [80]. Stigmasterol (**50**), comprising 11.85% of total sterols (265 mg/kg) in *C. spinosa*, was also identified in *C. ovata* and *C. formosana* [76,81,82]. Further compositional studies on *C. decidua* confirmed the presence of (**48**), (**44**), and (**50**) [83].

Taraxasterol (**51**) has also been reported from the leaves of *C. sepiaria* [84]. In addition, several glycosylated sterols have been identified, including β-sitosterol glucoside from *C. corymbose* [77], β-sitosteryl-glucoside-6′-octadecanoate from *C. spinosa* fruits [85], and α-spinasterol-3-O-β-D-glucopyranoside (**52**) from *C. spinosa* pericarp [86].

### 4.6. Essential Oil Constituents

Caper flowers and buds have characteristic aroma and flavour derived from hundreds of volatile compounds [65]. These include isothiocyanates, alcohols, aldehydes, ketones, esters, terpenes, and sulphur-containing compounds, many of which are released during steam distillation. Isothiocyanates, in particular, contribute to the distinctive pungent and sulphur-like aroma associated with caper essential oils [5]. Table 2 summarises the main components of Caper plant oils.

## 5. Pharmacological Activities

*Capparis* species exhibit diverse pharmacological effects, though most evidence to date comes from preclinical studies. Further well-designed clinical investigations are required to confirm their efficacy and safety.

### 5.1. Effect on Cardiovascular System

Several studies have highlighted the potential of *Capparis* species to modulate cardiovascular function through diverse mechanisms. Extracts from different plant parts—particularly aerial parts, roots, and fruits—have shown antihypertensive, cardioprotective, and antithrombotic activities in both in vivo and in vitro models, supporting their traditional use in cardiovascular disorders.

In normotensive anaesthetised rats, intravenous administration of the crude extract of *C. aphylla* (3–100 mg/kg) produced a dose-dependent reduction in mean arterial pressure. This effect was partly mediated by vasodilation and cardiac depression. Mechanistic studies indicated that the antihypertensive effect involved both endothelium-dependent nitric oxide pathways and endothelium-independent calcium channel blockade, with additional suppression of spontaneous atrial contractions in isolated atrial preparations. These findings suggest a dual mechanism that underpins the plant’s hypotensive action [92].

Similarly, the ethanolic extract of *C. cartilaginea* (1–10 mg/kg, i.v.) produced dose-dependent reductions in blood pressure and heart rate in anaesthetised rats. This effect appeared to be independent of cholinergic or adrenergic receptors, indicating a direct action on vascular smooth muscle and myocardium. Complementary isolated organ studies confirmed broad spasmolytic activity, as the extract inhibited norepinephrine- and K^+^-induced contractions in rabbit aorta, reduced force and rate of atrial contractions in guinea-pig atria, and relaxed smooth muscles in guinea-pig ileum and rat uterus [93].

Oral administration of an aqueous extract of *C. spinosa* fruits (150 mg/kg/day for 20 days) to spontaneously hypertensive rats produced a significant reduction in systolic blood pressure by day eight of treatment (*p* < 0.01), with a sustained effect throughout the dosing period. Interestingly, heart rate remained unchanged. Mechanistic observations included increased urinary output, enhanced glomerular filtration rate, and elevated urinary excretion of sodium, potassium, and chloride ions, suggesting a diuretic effect independent of the renin–angiotensin system [94].

The root bark of *C. erythrocarpus* demonstrated notable antihypertensive and lipid-lowering effects. Oral administration at doses of 20, 100, and 200 mg/kg/day for three months in Sprague Dawley rats resulted in a dose-dependent reduction in systolic blood pressure without affecting diastolic pressure. In addition, serum triglycerides, total cholesterol, and low-density lipoprotein LDL-cholesterol were significantly reduced, while High-density lipoprotein HDL-cholesterol increased. These effects, together with reduced food intake and body weight gain, highlight the potential of *C. erythrocarpus* in addressing hypertension associated with metabolic syndrome and obesity [95].

Methanol extracts of *C. decidua* fruits demonstrated moderate in vitro thrombolytic activity, achieving 23–32% clot lysis compared to the positive control streptokinase. Although preliminary, these findings support the traditional use of *C. decidua* in cardiac health and highlight its potential in preventing thrombotic cardiovascular events [96].

Finally, hydro-alcoholic extracts of *C. spinosa* significantly protected cardiomyoblast (H9c2) cells from doxorubicin-induced cardiotoxicity. Pretreatment with concentrations ranging from 25 to 200 µg/mL improved cell viability up to 90.9% of control levels (*p* < 0.001), reduced apoptosis, and enhanced antioxidant enzyme activity. These results point to a strong cardioprotective and antioxidant role of *C. spinosa*, particularly in mitigating chemotherapy-related oxidative stress and myocardial injury [96].

### 5.2. Anti-Cancer/Cytotoxic Activity

Extracts from *Capparis* species have shown notable anti-cancer and cytotoxic properties through multiple mechanisms, including apoptosis induction, cell cycle arrest, oxidative stress regulation, and modulation of epithelial–mesenchymal transition (EMT). These multi-targeted effects highlight the potential of these species as sources of bioactive compounds for cancer therapy.

The n-butanol extract of *C. spinosa* inhibited proliferation of human gastric carcinoma cells (SGC-7901) in a dose-dependent manner, with an IC_50_ of 31.5 µg/mL after 72 h, inducing 58.6–95.9% apoptosis at concentrations of 15–60 µg/mL. Mechanistic studies indicated calcium-mediated apoptotic pathways as a primary mechanism [97].

Hydroalcoholic extracts of *C. spinosa* rich in quercetin showed strong cytotoxic effects against human cervical carcinoma (HeLa), human breast adenocarcinoma (MCF-7), and human osteosarcoma (Saos) cancer cell lines, consistent with high antioxidant activity. Similarly, ethanolic extracts inhibited proliferation of HepG2 hepatocellular carcinoma cells (IC_50_ ≈ 1050 µg/mL), inducing morphological changes such as nuclear condensation and cell shrinkage indicative of apoptosis [98].

A methanolic leaf extract of *C. spinosa* demonstrated potent activity against human breast adenocarcinoma (MCF-7) breast cancer cells (IC_50_ = 3.6 ± 0.99 µg/mL) with a selective cytotoxic index of 1.17 compared to normal fibroblasts. Mechanistic studies revealed downregulation of surviving and upregulation of the tumour suppressor protein P27, suggesting strong involvement in apoptotic regulation [99].

Volatile isolates from *C. spinosa* subsp. *rupestris* were dominated by methyl isothiocyanate (80–90% of total volatiles) and showed potent cytotoxicity against MDA-MB-231 breast and T24 bladder cancer cells, with IC_50_ values of 3.81 µg/mL and 5.95 µg/mL, respectively, suggesting a key role of glucosinolate-derived compounds in anti-cancer activity [75].

Methanolic root extracts of *C. zeylanica* significantly inhibited proliferation and metastasis in breast cancer models, reducing cell viability of MDA-MB-231 and MCF-7 cells (IC_50_ = 19.1 µg/mL and 24.2 µg/mL, respectively). Treatment induced apoptosis, triggered S and G**_2_**/M phase cell cycle arrest, and modulated epithelial–mesenchymal transition (EMT) markers by downregulating transcription factors such as snail, slug, ZEB-1, and twist-1 while upregulating E-cadherin. Similarly, ethanol extracts of *C. cartilaginea* displayed moderate cytotoxicity against HCT-116, MCF-7, and RD cancer cell lines (IC_50_ = 39.1–102 µg/mL) [100].

Collectively, these findings demonstrate that the cytotoxic potential of *Capparis* species arises from a complex interplay of phytochemicals, including flavonoids, alkaloids, and glucosinolate derivatives. These compounds act through complementary pathways—inducing apoptosis, halting cell proliferation, and reducing metastatic potential. However, in vivo studies and clinical trials are still needed to confirm their efficacy, safety, and optimal formulations.

### 5.3. Anti-Diabetic Activity

Recent preclinical and clinical studies strongly support the antidiabetic potential of various *Capparis* species, such as *C. spinosa*, *C. decidua*, *C. zeylanica*, and *C. cartilaginea*, through multiple complementary mechanisms. These include stimulation of insulin secretion, protection and regeneration of pancreatic β-cells, modulation of glucose metabolism, and attenuation of oxidative stress and inflammation.

In a validated in vivo model, aqueous extract of *C. spinosa* significantly reduced fasting blood glucose in multi-low dose streptozotocin-induced diabetic mice. The extract suppressed basal endogenous glucose production and improved peripheral insulin sensitivity, as demonstrated using the euglycaemic hyperinsulinaemic clamp technique, indicating an insulin-sensitising mechanism of action [101].

*C. spinosa* root extracts (0.2–0.4 g/kg) improved hyperglycaemia, dyslipidaemia, and liver enzyme markers independently of insulin level changes [102]. Additionally, n-butanol fractions (200 mg/kg) alleviated diabetic neuropathy and restored neuronal antioxidant defences, neurotransmitter balance, and inflammatory mediator levels, likely due to their high polyphenol content [103].

*C. decidua* aqueous twig extract (250 mg/kg) improved fasting glucose and oxidative stress markers in diabetic rats, reversing pancreatic and hepatic histological damage [104]. Further studies also demonstrated that *C. decidua* seed oil and extracts exert antidiabetic effects [83]. Similarly, *C. zeylanica* methanolic fruit extract (200 mg/kg) reduced blood glucose by 35.5% and elevated circulating insulin by 81.8% after 28 days of treatment. In vitro studies on pancreatic β-cells (MIN6) confirmed its dose- and glucose-dependent insulin secretagogue activity [105].

*C. cartilaginea* leaf extract (200 mg/kg) exhibited significant antioxidant activity (DPPH IC_50_ = 187.36 µg/mL), improved glucose and lipid profiles, and increased HDL levels in alloxan-induced diabetic rats. Modest α-amylase inhibition (IC_50_ = 861.3 μg/mL) further suggested potential in regulating postprandial hyperglycaemia [15].

Importantly, a recent randomised, double-blind, placebo-controlled clinical trial involving 54 type 2 diabetic patients provided clinical evidence for the antidiabetic efficacy of *C. spinosa*. Participants receiving 400 mg caper fruit extract three times daily for two months showed significant reductions in fasting blood glucose (*p* = 0.037), glycosylated haemoglobin (HbA1c) (*p* = 0.043), and triglyceride levels, without adverse effects on liver or kidney function. This clinical confirmation strengthens the traditional use of *Capparis* species in diabetes management [106]. In another triple-blind, placebo-controlled trial, 30 patients with poorly controlled type 2 diabetes and metabolic syndrome received *C. spinosa* oxymel (hydroalcoholic fruit extract in oxymel, 10 mL thrice daily for 3 months). Treatment significantly reduced body weight and body mass index and prevented further increases in fasting and postprandial blood glucose, as well as hypertriglyceridaemia, compared to placebo. Although no significant improvement was observed in HbA1c or cholesterol levels, kidney and liver function remained unaffected, confirming the safety of the intervention [107].

These findings confirm that *Capparis* species modulate glucose homeostasis via complementary pathways—such as enhancing insulin secretion, reducing hepatic glucose output, improving peripheral glucose uptake, and mitigating oxidative stress. These effects are primarily attributed to bioactive compounds including polyphenols, flavonoids, and glycosides. However, further large-scale clinical trials are necessary to validate these outcomes and support the development of standardised therapeutic formulations.

### 5.4. Anti-Inflammatory and Pain-Relief Activities

Studies on the anti-inflammatory activity of *Capparis* species have been well documented, highlighting their potent immunomodulatory effects. These activities are largely attributed to their rich phytochemical profiles, which act through multiple pathways such as suppression of pro-inflammatory cytokines, mitigation of oxidative stress, and modulation of immune cell signalling.

*C. decidua* has shown notable anti-inflammatory effects. An ethanol extract of this species reduced inflammation in a carrageenan-induced ear oedema model in rats, demonstrating its potential for topical inflammatory conditions [108].

*C. heyneana* also exhibited transdermal anti-inflammatory and analgesic properties through its ethanolic extract, suggesting potential for dermal applications [5]. *C. zeylanica* has shown gastroprotective anti-inflammatory effects. A methanolic extract of its leaves significantly protected against ulcers caused by ethanol and indomethacin, reducing gastric volume and acidity by over 80% [109].

The most extensively studied species, *C. spinosa*, has been evaluated in relation to its systemic anti-inflammatory and immunological effects. Early studies showed that aqueous and ethanol extracts of *C. spinosa* significantly reduced inflammation in carrageenan-induced ear oedema in rats [108]. Aqueous extracts also decreased xylene-induced ear oedema and reduced pain in writhing and hot plate assays, effects attributed to polysaccharide content [110]. In a contact hypersensitivity (CHS) in vivo model, *C. spinosa* extracts modulated CD4^+^ T cell-mediated responses, reducing dermal inflammation, immune cell infiltration, and cytokine expression (IFN-γ, IL-17, IL-4), with the hexane fraction showing the strongest activity [111]. Additionally, *C. spinosa* has shown promise in managing systemic sclerosis-associated interstitial lung disease (SSc-ILD), reducing inflammation, skin fibrosis, and lung damage in a bleomycin-induced mouse model. It also inhibited fibrotic markers (α-SMA, TGF-β1) and suppressed fibroblast proliferation via mitogen-activated protein kinase **(**MAPK) pathway modulation [112].

In an ovalbumin-induced asthma model, *C. spinosa* fruit extract reduced eosinophil infiltration and pro-inflammatory cytokines (IL-4, IL-5, IL-13), comparable to dexamethasone. Another study on non-alcoholic steatohepatitis (NASH) demonstrated that *C. spinosa* extract, especially when combined with (**30**), synergistically reduced hepatic inflammation, fibrosis, and oxidative stress by modulating TGF-β/Smad signalling [113].*C. spinosa* also alleviated acute colitis in rats by reducing TNF-α, IL-6, and oxidative stress markers while enhancing antioxidant defences superoxide dismutase (SOD), catalase (CAT), and glutathione (GSH).

Significantly, preclinical findings are supported by limited but valuable clinical evidence. A randomised, double-blind, placebo-controlled trial in 30 women with refractory rheumatoid arthritis (RA) demonstrated that *C. spinosa* significantly improved both clinical and immunological outcomes. After three months of supplementation, patients showed reduced VAS pain scores (*p* = 0.0001), DAS28 (*p* = 0.007), ESR (*p* = 0.003), and CRP (*p* = 0.026). Immunological profiling revealed increased Treg cell frequency (*p* = 0.02) and Treg/Th17 ratio (*p* = 0.0015), together with reduced Th17 cell percentage (*p* = 0.04), indicating an immunomodulatory mechanism underlying the clinical benefit [114].

Similarly, *C. ovata* flower and fruit extracts exhibited dose-dependent anti-inflammatory effects in paw oedema models, though they lacked antithrombotic activity [115]. *C. cartilaginea* showed strong in vitro anti-inflammatory activity, inhibiting protein denaturation (93% at 500 µg/mL) and cyclooxygenase-1 (COX-1) (88% inhibition), though with lower potency than standard drugs like diclofenac [116]. Furthermore, our published work demonstrated that an extract from *C. cartilaginea* leaves and its isolated flavonoids, including (**32**), (**37**), and (**60**), significantly inhibited the activity of Matrix Metalloproteinase-9 (MMP-9) in lipopolysaccharide (LPS) stimulated macrophages, a key mechanism underlying its anti-inflammatory activity [60].

In terms of pain relief, certain *Capparis* species have shown promising results. *C. spinosa*’s polysaccharide-rich fraction reduced pain responses in hot-plate and writhing tests [108], while *C. ovata* methanol bud extract exhibited antinociceptive effects, suggesting central or peripheral pain modulation [117]. *C. sepiaria* root extracts displayed both anti-inflammatory and analgesic properties, and *C. zeylanica* leaf extracts increased pain thresholds in tail-immersion tests while inhibiting formalin-induced pain responses [118,119].

### 5.5. Anti-Infective Activities

The *Capparis* genus have exhibited notable anti-infective properties, including antibacterial, antifungal, antiviral, antiprotozoal, anthelmintic, and insecticidal activities. For instance, *C. decidua* has shown broad-spectrum antimicrobial activity through aqueous, chloroform, acetone, methanol, and ether extracts. Minimum inhibitory concentrations (MICs) as low as 0.028 µg/mL were observed against *Lactobacillus* spp., and isolated compounds outperformed standard antibiotics such as tetracycline and ciprofloxacin in growth inhibition assays [120]. Furthermore, the stem and flower extracts of *C. decidua* exhibited potent insecticidal and oviposition-deterrent activities against the pulse beetle *Bruchus chinensis*, a serious pest of stored food grains that causes significant damage to cowpea, gram, soybean, and other pulses [121].

*C. moonii* was reported to have tuberculostatic effects [92], while *C. sepiaria* exhibited dose-dependent anthelmintic activity comparable to albendazole [122]. The hydroalcohol extract of *C. sinaica* showed antiviral activity against herpes simplex virus (HSV) in vitro [123]. *C. spinosa* has been extensively studied for its anti-infective properties. Extracts from various parts of the plant demonstrated antibacterial, antifungal, antiprotozoal, molluscicidal, larvicidal, and antiviral effects. Notably, flavonoids such as (**29**) and (**30**) glycosides in methanol bud extracts were found to inhibit herpes simplex virus HSV-2 replication and enhance immune responses in human peripheral blood mononuclear cells [124]. Aqueous extracts suppressed dermatophytes (*Microsporum canis*, *Trichophyton violaceum*) at low concentrations [125], and chloroform extracts inhibited *Deinococcus radiophilus* growth [126].

Moreover, *C. spinosa* methanol and ethyl acetate extracts exhibited antimalarial activity against *Plasmodium falciparum* (IC_50_ = 0.50 µg/mL) without cytotoxicity to human cells [127], and seeds were shown to have anti- human immunodeficiency virus (HIV) and antifungal effects via protein constituents [128]. Extracts have also demonstrated synergistic effects with antibiotics, enhancing their efficacy against both susceptible and resistant *E. coli* strains [129]. Additional molluscicidal effects targeting *Biomphalaria alexandrina* and interference with parasite *Leishmania* major development have been attributed to lectins and toxic proteins present in the plant [130,131].

*C. stylosa* root extracts showed ichthyotoxic activity against *Channa punctatus* and demonstrated in vitro antibacterial properties, particularly with acetone and methanol solvents [132,133].

Recent investigations have underscored the antimicrobial potential of *Capparis spinosa*, particularly against *Acinetobacter baumannii* and *Streptococcus mutans*. Leaf and flower extracts showed concentration-dependent antibacterial and anti-virulence effects, with *S. mutans* displaying greater sensitivity (MIC = 16 mg/mL) than *A. baumannii* (MIC = 32 mg/mL). Gene expression analysis (qRT-PCR) demonstrated significant downregulation of virulence genes (*ompA*, *gtfB*) at sub-inhibitory levels (*p* < 0.001), suggesting interference with bacterial adhesion and biofilm formation [134].

Comparative studies on *C. spinosa* and *C. decidua* (both native to Pakistan) revealed stronger antibacterial activity in methanolic extracts of *C. decidua*. Against pathogens such as *Staphylococcus aureus*, *Escherichia coli*, *Bacillus subtilis*, and *Pasteurella multocida*, *C. decidua* extracts produced larger inhibition zones (up to 29.1 mm for *E. coli*) than *C. spinosa*. Notably, *C. spinosa* stem bark and *C. decidua* fruit extracts equally inhibited *B. subtilis* (26.8 mm), while *C. decidua* root extract significantly suppressed *P. multocida* (25.7 mm) [135].

*C. spinosa* has also been explored in biomedical applications. Hydrogels formulated with its leaf extract and sodium alginate exhibited a porous structure, potent antibacterial effects (notably against *E. coli* and *S. aureus*), and antioxidant properties. Cytotoxicity assays confirmed their compatibility with cell proliferation, indicating potential for wound healing and therapeutic use [136].

Further support for *C. decidua*’s antimicrobial efficacy was provided by methanol leaf extract tests against *S. aureus*, *B. cereus*, *S. typhi*, and *E. coli*. Disc diffusion assays revealed strong activity against *B. cereus* (21.21 ± 1.2 mm), comparable to ceftriaxone (21.21 ± 0.45 mm). MIC values demonstrated notable inhibition of *E. coli* (1.08 ± 5.81 mg/mL) and moderate inhibition of *S. aureus* (1.21 ± 3.1 mg/mL) [137].

Ethanol extracts of *C. spinosa* (leaves, roots, fruits) collected in inhibited six *Helicobacter pylori* strains, with leaf extracts showing the strongest activity (inhibition zones: 12.0–30.7 mm at 100 mg/mL) [138].

In addition, *C. spinosa* essential oil and its major components—methyl isothiocyanate, hexadecanoic acid, and limonene—exhibited insecticidal, antiplasmodial, and anti-leishmanial effects. The oil showed potent activity against *Aedes aegypti* larvae (LC_50_ = 21.6 μg/mL), *Plasmodium falciparum* (IC_50_ = 7.4 μg/mL), and *Leishmania major* amastigotes (IC_50_ = 9.1 μg/mL) [139].

### 5.6. Antioxidant Activity

Several species of the *Capparis* genus have demonstrated notable antioxidant properties through various in vitro and in vivo assays. For instance, the methanol stem extract of *C. saphylla* (30 mg/kg, orally) significantly improved antioxidant enzyme activity and prevented lipid peroxidation in streptozotocin-induced diabetic rats, possibly due to the modulation of glutathione levels, SOD, CAT, and glutathione peroxidase activities across the liver, heart, and kidney [140]. Likewise, powdered fruits of *C. decidua* reduced lipid peroxidation in erythrocytes, liver, and heart tissues in alloxan-induced rats, while enhancing CAT activity, suggesting hydrogen peroxide neutralisation [141]. Moreover, both aqueous and methanolic extracts of *C. decidua* stems mitigated fatty liver changes and liver enzyme elevations caused by carbon tetrachloride (CCl_4_), with effects comparable to silymarin. These effects were attributed to a complex mixture of phytochemicals such as alkaloids, flavonoids, tannins, sterols, saponins, cyanogenic glycosides, and coumarins [142]. The extract of *C. cartilaginea* leaves was also found to possess strong antioxidant activity, as evidenced by high total phenolic and flavonoid content, and potent free radical scavenging activity in DPPH and ABTS assays [60].

*C. spinosa* has been extensively studied for its antioxidant potential. Its aqueous alcohol extract exhibited radical scavenging activity in the DPPH assay [143], while its buds demonstrated antioxidant efficacy in vitro and in vivo, reducing UV-induced skin erythema in human volunteers. The activity was linked to flavonols ((**29**) and (**30**) derivatives) and hydroxycinnamic acids, including caffeic, ferulic, p-coumaric, and cinnamic acids [144]. Further tests confirmed that methanol extracts of the buds inhibited oxidative damage in multiple lipid peroxidation models, including liposome oxidation and UV-induced peroxidation [144]. Germano et al. [145] reported strong antioxidant action from the methanol bud extract against lipid peroxidation in rat liver microsomes and DPPH radical bleaching, primarily due to phenolic compounds.

In a dietary context, lactic-fermented *C. spinosa* buds in brine exhibited high total antioxidant potential (25.8 μmol Trolox equivalent), effectively inhibiting lipid peroxidation in a simulated gastric model. This was attributed to the synergistic action of rutin (13.76 mg per 8.6 g of capers), isothiocyanates, and total phenols [146]. Ethanol and ethyl acetate extracts of the fruit also showed strong DPPH radical scavenging activity, attributed to cappariside [147]. Other studies confirmed potent antioxidant activity of aqueous and ethanol fruit extracts using DPPH, ABTS, ferric thiocyanate, and metal chelation assays, showing efficacy comparable to butylated hydroxyanisole (BHA) and butylated hydroxytoluene (BHT), and greater than α-tocopherol [148]. Fractional extraction of the fruit further demonstrated that ethyl acetate fractions had the highest activity, followed by water, *n*-butanol, chloroform, and petroleum ether [147].

The antioxidant activity of *C. spinosa* leaves also varied by collection site across the trans-Himalaya, correlating with total flavonoid content [149]. Methanol fractions from aqueous extracts of *C. spinosa* showed antihepatotoxic effects in both in vivo and in vitro models [150], Similarly, ethyl acetate fractions rich in mono- and diglycosides were found to have stronger antioxidant effects than butanol fractions rich in polyglycosides [151].

### 5.7. Anti-Hyperlipidemia

The genus *Capparis*, including species such as *C. spinosa*, *C. decidua*, and *C. cartilaginea*, exhibits significant antihyperlipidemic activity, as demonstrated in multiple animal studies. *C. spinosa* aqueous extract (20 mg/kg) significantly reduced plasma triglycerides and cholesterol in both normal and streptozotocin-induced diabetic rats, with effects observed as early as four days post-treatment [152]. Similarly, a 50% ethanol extract of *C. decidua* administered for 30 days produced a dose-dependent decrease in total cholesterol, triglycerides, and LDL-C, while increasing HDL-C levels in diabetic rats [153]. *C. cartilaginea* leaf extract (200 mg/kg) also showed promising lipid-lowering effects, reducing LDL, total cholesterol, and triglycerides while elevating HDL after 14 days of treatment in alloxan-induced diabetic rats [15].

### 5.8. Other Pharmacological Activities

*C. masaikai* significantly improved oral moisture and oral conditions in humans [154]. Seeds of some species traditionally reduced heat and toxins, increased body fluids, and alleviated thirst. *C. cartilaginea* fruit extract demonstrated a significant increase in bone mineral density and improved bone turnover markers in osteoporosis-induced rats, suggesting potential for osteoporosis treatment [155].

Methanol extracts of *C. spinosa*, rich in (**29**) and (**30**), enhanced T lymphocyte proliferation and mitigated cyclophosphamide-induced myelosuppression in mice [156]. It also possesses antioxidant and photoprotective properties, reducing UV-induced skin erythema [144], and stimulates melanogenesis in melanoma cells [157]. Hepatoprotective effects of *Capparis* extracts against chemical-induced liver damage have been reported, supporting traditional liver therapies [150].

*C. decidua* extracts displayed central nervous system (CNS) depressant and anticonvulsant activities in animal models, including decreased motor activity, prolonged sleeping time, and reduced seizure incidence and severity in convulsion tests [158].

Additional studies reveal anti-allergic, bronchodilatory, analgesic, and antipyretic activities in various *Capparis* species, further expanding their therapeutic potential [159]. These findings collectively underscore the genus’s promise for diverse medicinal applications, warranting further research.

## 6. Materials and Methods

The literature was retrieved from PubMed, Scopus, ScienceDirect, and Google Scholar using the terms *Capparis*, *Capparis species*, *phytochemistry*, *ethnopharmacology*, and *pharmacological activity*. Searches included both classical references and recent studies, with coverage up to 2025. Only articles, books, and book chapters were considered. Non-scientific sources and duplicates were excluded. Reference lists of key publications were also screened to capture additional relevant studies.

## 7. Conclusions

This review highlights the diverse phytochemical profile and pharmacological potential of *Capparis* species, which are rich in alkaloids, flavonoids, glucosinolates, fatty acids, and sterols. These constituents are responsible for the reported antidiabetic, anti-inflammatory, antimicrobial, anti-cancer, cardiovascular, and antioxidant activities, supporting their traditional use. While preclinical findings are promising, clinical studies are needed to confirm the efficacy in humans. Future research should prioritise mechanistic studies, extract standardisation, and sustainable cultivation to facilitate the translation from ethnomedicine into clinical effective use and the integration of *Capparis* into evidence-based medicine.

## Figures and Tables

**Table 1:** Summary of selected *Capparis* species, their geographic occurrence, and reported traditional medicinal applications based on ethnobotanical sources.

Species	Common Name	Region	Part Used	Traditional Uses	Reference
*Capparis acutifolia* Sweet	Chinese caper	China, Taiwan, Bhutan, India, Thailand, Vietnam	Roots	Rheumatic arthralgia; abdominal pain	[12,13]
*Capparis brevispina* DC.	Indian caper	South India, Sri Lanka	Not specified	Stomachic; tonic; wound healing; fever; hepatoprotective	[14]
*Capparis cartilaginea* Decne.	Cartilage caper;	NE and E Africa, Arabian Peninsula, W. Asia, Indian Subcontinent	Fruit; Leaf; Stem/Shoots; Root	Rheumatism, arthritis, skin inflammation, wounds, bruises, childbirth, earache, diabetes, antiseptic	[15,16,17]
*Capparis decidua* (Forssk.) Edgew.	Bare caper	N and Tropical Africa, W. Asia, Indian Subcontinent	Fruit; Bark; Root; Stem; Leaf; Flower buds	Rheumatism, arthritis, asthma, cough, toothache, GI disorders, diabetes, cardiac issues, skin problems, antidiabetic, antioxidant	[18,19,20]
*Capparis divaricata* Lam.	Spreading caper.	India, Sri Lanka	Bark; Leaf	Analgesic, diuretic, antiulcer, aphrodisiac, skin eruptions, insect bites, infertility	[21,22]
*Capparis flavicans* Kurz	Hedge caper or wild caperbush	India, Cambodia, Myanmar, Thailand	Leaf	Galactagogue	[23]
*Capparis grandis* L.f.	Grand caper or tree caper	India, Sri Lanka, Myanmar, Thailand	Leaf; Bark; Root; Flower	Asthma, skin eruptions, wounds, insect bites, blood tonic, sterility, paralysis	[24]
*Capparis moonii* Wight.	Large caper and moon’s caper.	South India, Sri Lanka	Fruit; Seed	Asthma, cough, pulmonary tuberculosis, weakness	[25,26]
*Capparis rheedei* DC.	Rheed’s wild caper	Central and South America, Caribbean	Not specified	Diuretic, sedative, skin problems, spasms, emmenagogue	[27]
*Capparis sepiaria* L.	Wild caper bus	Africa, China, Indian Subcontinent, Indochina, Malesia, Australia	Seed; Leaf; Root; Stem; Root bark; Flower	Digestive disorders, diabetes, respiratory issues, skin diseases, blood purifier, tonic, antipyretic, anti-inflammatory, gout	[28,29]
*Capparis spinosa* L.	Caper bush	Mediterranean, Africa, Europe, Middle East, Asia, Pacific	Flower buds, Fruits, Leaf, Branch tips, Shoots, Root, Root bark	Pickled condiment; GI disorders; rheumatism; gout; haemorrhoids; fever; liver and kidney issues; headache; toothache	[30,31]
*Capparis spinosa var. aegyptia* (Lam.) Boiss.	Egyptian caper	N. Africa, Middle East, E. Mediterranean	Root bark; stem bark; fruit	Anti-inflammatory; diuretic; rheumatism; arthritis; gout; hypertension; malaria; GI problems	[8,32]
*Capparis spinosa subsp. himalayensis* (Jafri) Fici	-	Himalayan region, Central Asia, Caucasus, China	Leaf, Fruits, Root, Root bark, Part not specified	Rheumatism; gout; palsy; joint pain; sores; paralysis; toothache; intestinal worms	[33,34]
*Capparis spinosa subsp. Rupestris* (Sm.) Nyman	Rock caper	Mediterranean, S. Europe, N. Africa, W. Asia, N. S. America	Plant	Stomach ailments	[6,34]
*Capparis spinosa var. ovata* (Desf.) Sm.	-	Algeria, Libya, Morocco, Italy, Tunisia	Root; Part not specified	Digestive disorders; respiratory problems; anti-inflammatory; headache; snakebite	[6,35,36]
*Capparis spinosa var. canescens* Coss.	-	W. Asia, Arabian Peninsula	Root; Root bark; Part not specified	Rheumatism; respiratory issues; diuretic; expectorant; snakebite antidote	[37]
*Capparis tomentosa* Lam.	Woolly caper bush or African caper	Tropical Africa, Arabian Peninsula	Fruit, Leaf, Stem, Bark, Root, Root ashes, Root bark	Rheumatism; reproductive health; respiratory and GI disorders; malaria; diabetes; psychiatric conditions; skin infections; snakebite	[38,39,40]
*Capparis zeylanica* L.	Ceylon caper	Tropical Asia, Malesia, Indochina	Leaf, Root bark, Part not specified	Anti-inflammatory; analgesic; febrifuge; helminthic infections; GI issues; immune disorders; paralysis; rheumatism	[41,42]

Abbreviations: GI = gastrointestinal.

**Table 2:** Essential oil composition of Caper plants.

Class	Species	Part Used	Compounds	Reference
Isothiocyanates	*C. spinosa*	Leaves	Methyl isothiocyanate, Ethyl isothiocyanate, Isopropyl isothiocyanate, Butyl isothiocyanate, Isobutyl isothiocyanate	[87]
		Leaves and flower buds	Methyl isothiocyanate, Butyl isothiocyanate, Isobutyl isothiocyanate, Benzyl isothiocyanate, Benzyl isocyanide	[88]
	*C. zeylanica (syn. flexuosa)*	Trunk bark, leaves, and pod peel	3-Methyl-3-buteneisothiocyanate, Butyl isothiocyanate	[89]
	C. ovata Desf. var. canescens	Buds and leaves	Methyl isothiocyanate, Isopropyl isothiocyanate, butyl isothiocyanate, isobutyl isothiocyanate	[90]
	C. grandis	Roots	4,5,6,7-Tetrahydroxydecyl isothiocyanate	[50]
	*C. cartilaginea*	Leaves	Isopropyl isothiocyanate, *(E*)-1-Isothiocyanato-2-butene, 2-Butyl isothiocyanate, Isobutyl isothiocyanate	[91]
Terpenoids	*C. sepiaria*	Leaves	α-Amyrin, Erythrodiol, Taxasterol	[84]
	*C. spinosa*	Leaves	Eucalyptol, Linalool, Sabinol, (*E*)-*p*-Mentha-2,8-dien-1-ol, (*Z*)-p-Mentha-2,8-dien-1-ol, Camphor, Karahanaenone, Menthone, Pinocarvone, Isomenthone, Neomenthol, Terpinen-4-ol, *p*-Cymen-8-ol, *α*-Terpineol, Nerol, (*Z*)-Carveol, Pulegone, Carvone, Geraniol, Perilla alcohol, Caryophyllene oxide, *α*-Bisabolol oxide B	[87]
	*C. cartilaginea*	Leaves	Myrcene, *p*-Cymene, Limonene, Eucalyptol, γ-Terpinene, Linalool, Camphor, 4-Terpineol, *p*-Cymen-9-ol, α-Terpineol, *O*-Methylthymol, Cumin aldehyde, Piperitone, Thymol, α-Terpinyl acetate	[91]
Volatile Acids	*C. spinosa*	Flower buds	Octanoic acid, Nonanoic acid, Decanoic acid.	[88]
	*C. cartilaginea*	Leaves	Dodecanoic acid	[91]
Volatile Esters	*C. spinosa*	Leaves	2-Propenyl hexanoate, Methyl 2,6-cresote (methyl 2,6-dimethylbenzoate), Massoia lactone, (*Z*)-3-Hexenyl benzoate, Isopropyl tetradecanoate, 2-Phenylethyl benzoate	[87]
		Leaves and flower buds	Butyl 2-propenoate, Methyl benzoate, Methyl octanoate, Linalyl acetate, Isoamyl benzoate, Methyl laurate, Ethyl benzoate	[88]
	*C. cartilaginea*	Leaves	(*Z*)-3-Hexen-1-yl benzoate	[91]
	*C. ovata* Desf. var. *canescens*	Buds and leaves	Ethyl 2-hydroxypropionate, Hexyl acetate, 2-Phenylethyl acetate, Benzyl isovalerate, Methyl hexadecanoate	[90]
Volatile Ketones	*C. spinosa*	Leaves	3-Heptanone, 2-Heptanone, 3-Methyl-2-cyclohexen-1-one, 1-Octen-3-one, 6-Methyl-5-hepten-2-one, 2-Octanone, Acetophenone, 2-Nonanone, (*E*,*E*)-3,5-Octadien-2-one, 6-Methyl-3,5-heptadien-2-one, 2-Nonen-4-one, 3-Nonen-2-one, Benzophenone	[87]
		Leaves, aerial parts	6-Methyl-5-hepten-2-one, 3,5-Octadien-2-one, Neryl acetone.	[88]
	*C. ovata* Desf. var. *canescens*	Buds and leaves	Cyclohexanone, 2-Heptanone, 6-Methyl-5-hepten-2-one, (*E*,*E*)-3,5-Octadien-2-one, Isophorone (3,5,5-trimethyl-2-cyclohexen-1-one), Karahanaenone (2,2,5-trimethyl-4-cycloheptene-1-one), Frambinone [4-(4-hydroxyphenyl)-2-butanone], Zingerone [4-(4-hydroxy-3-methoxyphenyl)-2-butanone], 4-(3-hydroxy-2-methoxyphenyl)-2-butanone	[90]
Volatile aldehydes	*C. spinosa*	Leaves	(*E*)-2-Hexenal, Octanal, Nonanal, Decanal, 2-Phenyl-2-butenal, 4-Methyl-2-phenyl-2-pentenal, 5-Methyl-2-phenyl-2-hexenal	[87]
	*C. ovata* Desf. var. *canescens*	Buds and leaves	Hexanal, Furfural, (*E*)-2-Hexenal, Heptanal, Benzaldehyde, Phenylacetaldehyde, 2,4-Dimethylbenzaldehyde, Cinnamic aldehyde (3-phenyl-2-propenal), Vanillin (4-hydroxy-3-methoxybenzaldehyde)	[90]
Volatile Alcohols	*C. spinosa*	Leaves	(*Z*)-3-Hexen-1-ol, 1-Hexanol, 2,4-Dimethyl-3-heptanol, 1-Heptanol, 1-Octen-3-ol, 6-Methyl-5-hepten-2-ol, 3-Ethyl-hexanol, 2-Octen-1-ol, 1-Nonen-4-ol, 1-Octanol, 1-Nonanol, 1-Decanol, 1-Dodecanol, 1-Tetradecanol, 1-Hexadecanol (*Z*)-3-Hexen-1-ol, 1-Hexanol, 2,4-Dimethyl-3-heptanol, 1-Heptanol, 1-Octen-3-ol, 6-Methyl-5-hepten-2-ol, 3-Ethyl-hexanol, 2-Octen-1-ol, 1-Nonen-4-ol, 1-Octanol, 1-Nonanol, 1-Decanol, 1-Dodecanol, 1-Tetradecanol, 1-Hexadecanol	[87]
Sulphur-Containing Compounds	*C. spinosa*	Leaves	Dimethyl disulfide, Dimethyl trisulfide, Dimethyl tetrasulfide, Dimethyl pentasulfide, Cyclic octatonic sulphur	[87]

## Data Availability

The data presented in this study are available on request from the corresponding author.

## Supplementary Materials

The following supporting information can be downloaded at: https://www.mdpi.com/article/10.3390/molecules30183705/s1.
