# Supplementary material for: Capparis L. (Capparaceae): A Scoping Review of Phytochemistry, Ethnopharmacology and Pharmacological Activities
Source: Molecules. 2025 Sep 11;30(18):3705. doi: 10.3390/molecules30183705 (PMC12472821; doi:10.3390/molecules30183705)
Supplement: Supplementary file 1 [file molecules-30-03705-s001.zip › molecules-3835848-supplementary.pdf]

Species	Photographer	Source (link)	Licence
Capparis acutifolia	Cynthia Chan	https://www.inaturalist.org/photos/501852882	CC BY-NC-ND 4.0
Capparis spinosa var. aegyptia	Theodore	https://www.inaturalist.org/photos/182596067	
Capparis brevispina	V.Arun	https://www.inatu.../photos/476158763	
Capparis rheedei	Suraj Malik	https://www.inaturalist.org/photos/484431189	
Capparis cartilaginea	Judy Flatt	https://www.inaturalist.org/photos/154919808?size=large	
Capparis decidua	Sonu Kumar	https://www.inaturalist.org/photos/373914967	
Capparis divaricata	Siddarth Machado	http://inaturalist.org/photos/42832258	
Capparis flavicans	Kasorn Klankhunthod	https://www.inaturalist.org/observations/106849041	
Capparis grandis	Jayant M Deshpande	https://www.inaturalist.org/observations/158471283	
Capparis spinosa subsp. himalayensis	Nomadash	https://www.inaturalist.org/observations/117297432	
Capparis zeylanica	Abhilare	https://www.inaturalist.org/observations/37093027	

Capparis sepiaria	Yvettevw	https://www.inaturalist.org/observations/37093027	
Capparis spinosa var. canescens	Marc Deckert	https://www.inatu.../photos/514110987	
Capparis moonii	S.MORE	https://www.inaturalist.org/observations/38149154	
Capparis spinosa var. ovata	Ori Fragman-Sapir	https://inaturalist.lu/photos/266059350	
Capparis spinosa	Ori Fragman-Sapir	https://www.inaturalist.org/observations/154009552	
Capparis tomentosa	Darren Obbard	https://inaturalist.lu/photos/317800150	
Capparis spinosa subsp. Rupestris	Guta	https://www.inaturalist.org/photos/560517204	
